# Microscopes of American Manufacture

**Published:** 1855-09

**Authors:** 


### MICROSCOPES OF AMERICAN MANUFACTURE.

Physicians frequently apply to us for information respecting the best form of the microscope, and the place for obtaining them. We are happy to refer our readers to one of American manufacture, indorsed in the following terms by our friend, Professor Silliman, whose opinions on such a subject are of the highest authority:

"A few years since—only five or six—it was a thing impossible to obtain in the United States a microscope of any value of domestic manufacture. Thanks to the genius of Spencer and the Messrs. Grunow of New Haven, this is now quite possible. The merit of Mr. Spencer in this department of optical art, the clearness of definition, power of illumination, absence of color and breadth of angular aperture, in his higher powers, are well known, and have been long since acknowledge, even by his European competitors. In the New York crystal palace exhibition, the first prize for micrscopes was awarded to Mr. Spencer, the second to Messrs. Grunow. Since that time the latter makers have steadily advanced in the excellence of all parts of their instruments. Their form of stand for steadiness, ease of management. simplicity and economy, is unrivalled; and the movable stage with universal motion in one plane by one lever, improved and admirably executed by them, is now universally adopted in the best American instruments. Their brass work is perfect in all respects. One of the most experienced microscopists in the United States writes as follows of the optical value of their objectives: 'I have tried the $\frac{1}{8}$th objective, and it compared so well with my Spencer $\frac{1}{8}$th that I consider them of equal vualue. I have also a $\frac{1}{4}$th inch objective made by them which works quite as well as the best English glasses. I need not tell you how much I rejoice that so worthy a man as Mr. Grunow has succeeded so well as an optician. His Student's Microscope I have been familiar with for some time. It is a source of congratulation that our students of medicine can now obtain the means of pursuing microscopic studies at a reasonable cost, and without the vexatious delays we have experienced in importing European instruments.'"

